# Single-nucleotide polymorphism identification and genotyping in *Camelina sativa*

**DOI:** 10.1007/s11032-015-0224-6

**Published:** 2015-01-21

**Authors:** Ravinder Singh, Venkatesh Bollina, Erin E. Higgins, Wayne E. Clarke, Christina Eynck, Christine Sidebottom, Richard Gugel, Rod Snowdon, Isobel A. P. Parkin

**Affiliations:** 1 Agriculture and Agri-Food Canada, 107 Science Place, Saskatoon, S7N 0X2 Canada; 2 School of Biotechnology, Sher-e-Kashmir University of Agricultural Sciences and Technology of Jammu, Jammu, 180 009 JK India; 3 National Research Council Canada, 110 Gymnasium Place, Saskatoon, S7N 0W9 Canada; 4 Plant Gene Resources Canada, 107 Science Place, Saskatoon, S7N 0X2 Canada; 5 Department of Plant Breeding, Justus Liebig University, Heinrich-Buff-Ring 26-32, 35392 Giessen, Germany

**Keywords:** *Camelina sativa*, Reduced representation, SNP, Genetic mapping, Diversity, Polyploidy

## Abstract

*Camelina sativa*, a largely relict crop, has recently returned to interest due to its potential as an industrial oilseed. Molecular markers are key tools that will allow *C. sativa* to benefit from modern breeding approaches. Two complementary methodologies, capture of 3′ cDNA tags and genomic reduced-representation libraries, both of which exploited second generation sequencing platforms, were used to develop a low density (768) Illumina GoldenGate single nucleotide polymorphism (SNP) array. The array allowed 533 SNP loci to be genetically mapped in a recombinant inbred population of *C. sativa*. Alignment of the SNP loci to the *C. sativa* genome identified the underlying sequenced regions that would delimit potential candidate genes in any mapping project. In addition, the SNP array was used to assess genetic variation among a collection of 175 accessions of *C. sativa*, identifying two sub-populations, yet low overall gene diversity. The SNP loci will provide useful tools for future crop improvement of *C. sativa*.

**Electronic supplementary material:**

The online version of this article (doi:10.1007/s11032-015-0224-6) contains supplementary material, which is available to authorized users.

## Introduction

*Camelina sativa* (L. Crantz) is a species from the highly diverse Brassicaceae family, which contains a number of economically important oilseed crops (Bailey et al. 2006). Recently *C. sativa* has garnered interest as a possible non-food oilseed platform for bioproducts and biofuels, which could complement its crop relatives from the Brassiceae tribe. As a crop *C. sativa* benefits from a short generation time and innate biotic and abiotic stress tolerance. Furthermore, it is amenable to similar production practices as the widely grown oilseed crop *Brassica napus* (Séguin-Swartz et al. 2009). These various attributes would allow *C. sativa* to be grown both in more Northern latitudes and in more arid areas than *B. napus*. The potential of *C. sativa* as a crop for the Canadian Prairies has already been established (Gugel and Falk 2006). Although harbouring many positive traits *C. sativa* has not been grown extensively since the 1950s and to ensure its establishment as a viable crop, improvements need to be made to seed size, yield traits, oil content and disease tolerance.

The recent publication of the genome sequence of *C. sativa* represented a significant advance for further research targeting this promising oilseed (Kagale et al. 2014). Previous efforts in molecular genetic analyses of *C. sativa*, have mostly focused on identifying the range of available genetic diversity within the species. Vollmann et al. (2005) observed low levels of genetic diversity in 41 *C. sativa* accessions studied through RAPD genotyping. More variation was suggested when studying a collection of 53 *C. sativa* accessions using AFLP markers; however, the study was somewhat biased due to a limited geographical sampling area (Ghamkhar et al. 2010). These studies should prove invaluable for identifying novel variation for useful traits; however, they provided no genome context for the published marker data. In addition the previously available genetic map for *C. sativa* (Gehringer et al. 2006) was derived using AFLP technology which precludes comparison either within or across species and such markers are recalcitrant to conversion to locus specific markers, an essential prerequisite for marker-assisted selection.

The development of robust genetic markers allows genomic regions controlling traits of interest to be tagged and followed in marker-assisted selection, which can expedite crop improvement strategies (Collard and Mackill 2008). In addition, molecular markers allow comprehensive assessment of available genetic variation within a species leading to the identification of novel alleles for traits of interest. Single nucleotide polymorphisms (SNP) are valued as genetic markers in plants due to their generally uniform distribution across the genome, relative abundance and their ability to be used on multiple platforms, including massively parallel array systems (Ganal et al. 2009). Genome-wide SNPs in *C. sativa* could also be anchored to the genome sequence allowing rapid identification of candidate genes for traits of interest and providing genomic substrates for targeted marker development. The genome sequence of *C. sativa* uncovered a relatively undifferentiated hexaploid genome with strong conservation of sequence identity between the three subgenomes (Kagale et al. 2014). This genome structure hampers the development of robust single copy SNP loci assayed through standard procedures, which are dependent upon specific hybridization to short oligonucleotide sequences, thus confounded by the presence of duplicated loci. In particular in the absence of a genome sequence, development of SNP loci that identify true intra-locus polymorphisms requires additional processing steps to ensure their specificity.

The current research describes two alternative methods for SNP discovery in *C. sativa* in the absence of a genome sequence, the development of an Illumina GoldenGate SNP array, the generation of a SNP map for *C. sativa* subsequently anchored to the genome sequence, and assessment of genetic variation in a wide collection of *C. sativa* accessions. The linkage map allowed conserved syntenous blocks common to all Brassicaceae species to be identified within the *C. sativa* genome (Schranz et al. 2006), providing a useful platform for identifying candidate genes for traits of interest. The SNP loci provide an excellent basis to establish genome-based improvement of this emerging industrial oilseed crop.

## Materials and methods

### Plant materials

In total four *C. sativa* lines were used for SNP discovery using two different approaches. *C. sativa* lines 31471-03 and 33708-06, are progenitor lines of 36011 and 36012 that show a differential response to sclerotinia infection as described in Eynck et al. (2012). Plants of these two lines were grown in a greenhouse and tissue collected from whole seedlings 2 weeks after germination. RNA was extracted using the Qiagen RNeasy mini kit according to the manufacturer’s protocol (Qiagen Inc., Toronto, ON, Canada). cDNA was synthesized from five µg of total RNA according to Sharpe et al. (2013). Advanced inbred lines of the old German cultivars Licalla and Lindo (DSV Seeds, Lippstadt, Germany), which were used to derive a recombinant inbred (RI) population as described in Gehringer et al. (2006), were used to develop reduced representation genomic next generation sequencing libraries. Plant tissue was collected from greenhouse grown plants at approximately 2 weeks after germination and DNA extracted according to Sharpe et al. (1995).

For diversity analysis, a collection of 178 accessions was obtained from Plant Gene Resources of Canada (PGRC, Saskatoon, SK, Canada; http://pgrc3.agr.gc.ca) (Supplementary Table 1). DNA was extracted from freeze dried tissue of young leaves using a cetyltrimethylammonium bromide (CTAB) based method (Murray and Thompson 1980).

### 3′ cDNA library construction and Roche 454 next generation sequencing

3′ biased 454 Roche libraries were generated from cDNA digested with *Aci*I (20 U) for 1 h at 37 °C. Small fragments were removed by hybridizing the digested cDNA to AMPure beads (Agencourt Bioscience Corporation, Beverly, MS, US) for 5 min at room temperature, washing with 70 % ethanol and eluting the cDNA in 10 mM Tris Buffer. The adaptor was prepared by annealing 100 pmol of oligo A1 (CCATCTCATCCCTGCGTGTCTCCCACTCAGCAT) and 100 pmol of oligo A2 (CGATGCTGAGTCGGAGACACGCAGGGATGA) in annealing buffer (1 mM Tris–HCl pH 8, 15 mM MgCl_2_, 15 mM NaCl, 0.1 mM spermidine) at 55 °C for 5 min and then allowing the mixture to return to room temperature. Purified cDNA was hybridized with 25 μL M-270 streptavidin beads (Dynabeads, Life Technologies Inc., Burlington, ON, Canada) for 20 min at room temperature. The adaptor (5 pmol) was ligated to the immobilized library for 20 min at room temperature, followed by a fill-in reaction using Large Fragment *Bst* Polymerase (24 U, NEB, Whitby, ON, Canada) for 20 min at 42 °C. The single-stranded library was eluted from the beads using 0.1 N NaOH and neutralized in Qiagen PBI buffer containing NaOAc pH5.2. The neutralized, single-stranded library was cleaned using a Qiagen MinElute Kit according to the manufacturer’s protocol. The libraries were sequenced on a Roche 454 GS FLX sequencer in the DNA Technologies Laboratory at National Research Council, Saskatoon.

### Reduced representation library preparation and Illumina sequencing

Ten microgram of genomic DNA from Lindo and Licalla were digested to completion with *Eco*RI (4 U/μg) for 12 h at 37 °C. The digested DNA was separated in 0.7 % agarose (1× TAE) for 2 h at 110 V, fragments between 2 and 4 Kb in length were excised from the gel and eluted from the agarose using QIAquick gel extraction kit. The eluted DNA was then used to generate a reduced representation library with the Illumina paired-end sample preparation kit according to the manufacturer’s protocol, with a final insert size of approximately 300 bp (Illumina Inc., San Diego, CA, USA). The libraries for each line were sequenced for 101 cycles from each end of the insert on an Illumina Genome Analyser IIx in the DNA Technologies Laboratory at National Research Council, Saskatoon.

### SNP discovery and array development

The 454 transcriptome data was processed and analysed using SeqMan NGen v2.1.0. The raw data were trimmed for quality and adapter sequence. Sequences from line 33708-06 were assembled de novo using the following parameters: match Size 19, match Spacing 75, minimum match percentage 95, match score 10, mismatch penalty 25, gap penalty to generate 25, maximum gap 15 and expected coverage 100. The filtered sequences from line 31471-03 were reference mapped to the assembled contigs using the same parameters as for the de novo assembly except that the minimum match percentage was increased to 98. Nucleotide variation was identified in NGen using default parameters. The resultant list of SNPs was filtered as described in "Results".

The Illumina genomic data were imported into CLCBio Genomics Workbench v4. for subsequent analysis. The sequences were trimmed for quality, length and presence of adapter sequence. The sequence data for Lindo were assembled de novo with default parameters, specifically with a sequence similarity of 0.8 over 0.5 of the read length. The sequence data for Licalla were referenced mapped to Lindo using default parameters (as above), with only unique matches being considered. SNP variants were called with a minimum variant frequency of 35 % and a predicted genome ploidy level of three, since it had been previously suggested that *C. sativa* was an ancient hexaploid (Hutcheon et al. 2010). Potentially useful SNPs were filtered using custom Perl scripts as described in the "Results".

The sequences containing potential SNPs along with 100 bp of flanking DNA were submitted to the Illumina^®^ Assay Design Tool (ADT) to generate an ADT score; those SNPs falling below 0.6 were rejected. The final selection of 768 SNP loci was submitted to Illumina to generate the custom pooled oligo set (OPA).

### Genetic mapping

DNA was extracted from Lindo, Licalla and 180 lines of the RI mapping population according to Murray and Thompson (1980). Forty-six SSR loci had previously been mapped on the same population (unpublished data). DNA was quantified with the Quant-it Picogreen dsDNA Assay Kit (Life Technologies Inc., Burlington, ON, Canada) and 200 ng was hybridized to the *C. sativa* Illumina GoldenGate array according to the manufacturer’s instructions. Subsequently the arrays were scanned using an Illumina HiScan. The SNP data were analysed and the genotypes for each line called using the Genotyping module of the GenomeStudio software. The genetic linkage map was generated using Mapmaker v3 with a LOD score of 3.0 (Lander et al. 1987). The map order was checked manually for the presence of double crossovers, which might indicate incorrectly placed loci, and the final map distances were generated using the Kosambi mapping function. The map was drawn using MapChart v2.2 software (Voorrips 2002).

### Population genetic analyses

STRUCTURE v2.3.4 was used to analyse the population structure (Pritchard et al. 2000). To estimate the posterior probabilities (*qK*) a 100,000 burn-in period was used, followed by 100,000 iterations using a model allowing for admixture and correlated allele frequencies with no a priori location or population information. At least 10 independent runs of STRUCTURE were performed by setting K from 1 to 10, with 10 replicates for each *K*. The ∆*K* was calculated for each value of *K* using Structure Harvester (Evanno et al. 2005; Earl and vonHoldt 2012). A line was assigned to a given cluster when the proportion of its genome in the cluster (*qK*) was higher than a standard threshold value of 70 %. For the chosen optima value of *K*, membership coefficient matrices of replicates from STRUCTURE were integrated to generate a *Q* matrix using the software CLUMPP (Jakobsson and Rosenberg 2007) and the STRUCTURE bar plot was drawn using the DISTRUCT software (Rosenberg 2004).

Statistics including gene diversity, PIC value and allele frequency for each locus were calculated using Powermarker v3.25 (Liu and Muse 2005). AMOVA was performed using Arlequin version 3.5.1.3 (Excoffier and Lischer 2010). A phylogenetic tree was constructed using the unweighted Neighbour-Joining tree implemented in Darwin (http://darwin.cirad.fr/darwin). Bootstrap support for this tree was determined by resampling loci 1000 times.

## Results

### SNP discovery and array design

Two approaches, both using next generation sequencing (NGS), were adopted to identify SNPs in the *C. sativa* genome. The first involved the development and sequencing of cDNA libraries that were targeted to capture the 3′ end of expressed transcripts and the second approach used reduced representation through restriction digestion and size selection to limit the regions of the genome that were being assayed.

The 3′ biased cDNA libraries were sequenced using Roche 454 and 956,538 high quality sequences were generated from line 33708-06 and 586,982 for line 31471-03. Since no reference genome sequence was available for *C. sativa*, a de novo assembly was generated for line 33708-06, which resulted in 582,229 reads (60.9 %) being assembled into 47,313 contigs with an average length of 425 bp. Seventy-four percent (435,016) of the reads from line 31471-03 were reference mapped to the assembled contigs with a fivefold average coverage. Nucleotide variation was identified using a depth cut-off of 3 and a variant percentage of 30, which identified 8,037 SNPs (2,683 contigs) and 21,537 insertion/deletions (6,509 contigs). Due to the anticipated polyploid nature of the genome and the desire to generate locus-specific SNPs, further filtering required both the reference and the alternate base to be represented in 100 % of the reads. This significantly reduced the potential number of useful SNPs to 426 (5 % of the observed variation). Screening for SNPs with sufficient flanking sequence that also passed Illumina’s quality check for probe design (ADT score >0.6) identified 252 SNP loci, which were submitted for Illumina GoldenGate array design.

The reduced representation genomic libraries were sequenced on the Illumina GAIIx platform and the resultant data for each line are shown in Supplementary Table 2. Eighty-two percent of the Lindo reads (84,331,454) were de novo assembled using CLCBio Genomics Workbench to generate 288,946 contigs (≥200 bp), with an average length of 511 bp covering 147.7 Mb of genome sequence. The data from Licalla was referenced mapped to the Lindo contigs, resulting in alignment of 46,922,482 reads to 260,431 contigs. SNP detection using CLCBio identified 234,838 SNP positions with a single variant base in Licalla at a depth of at least 8 reads and a variant percentage greater than 35 %. In order to reduce the impact of duplicate loci only SNPs where the reference and alternate base showed no variation were further processed. This reduced the number of available SNP positions to 48,421 (20.6 % of possible variation). In addition, SNPs were further restricted by selecting those with 100 bp of flanking sequence and which contained no additional SNPs, reducing the available SNPs to 6,686 in 4,919 contigs. These SNPs were submitted to Illumina’s Assay Design Tool and only those with a score of >0.6 were considered further. In an attempt to select SNPs across the genome, inferred synteny with *Arabidopsis thaliana* was exploited. The sequence of each contig with potentially useful SNPs was aligned to the *A. thaliana* genome using BLASTN (*E* value cut-off of 1E−12). Approximately 50 % of the contigs (2,448) were homologous to 1,878 annotated *A. thaliana* genes. A subset of SNPs were selected for the array design from contigs that potentially covered the expanse of the *A. thaliana* genome. This represented 288 SNPs that were positioned in contigs with homology to 64, 58, 48, 47 and 61 *A. thaliana* genes on chromosomes one to five, respectively. Since genic SNPs can be less robust due to the influence of unidentified homologues, 228 SNPs were chosen randomly from those assumed to be intergenic. Including SNPs designed from the 3′ cDNA analyses a total of 768 SNPs were submitted for Illumina GoldenGate array design (Supplementary Table 3).

### Genetic linkage map for *Camelina sativa*

**Fig. 1:**
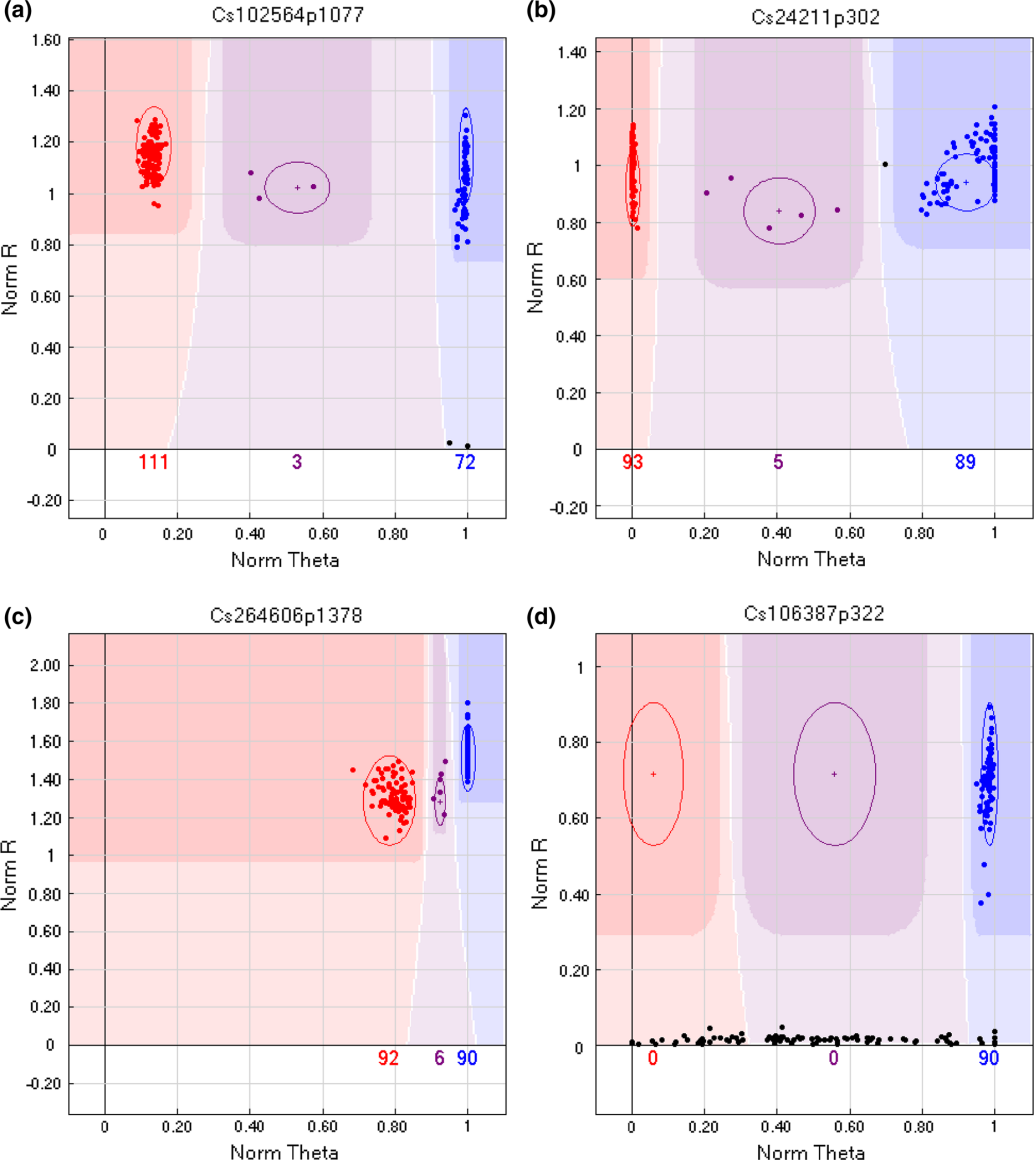
GenomeStudio images of SNP markers segregating in the RIL Population. **a** SNP showing typical 3 cluster segregation pattern; **b** SNP where the hybridization of one allele was affected perhaps by the presence of an additional SNP in the flanking sequence; **c** SNP with extremely low cluster separation, requiring manual editing of the clusters; and **d** dominant SNP for which only one allele could be scored

A recombinant inbred (RI) population derived from a cross between Lindo and Licalla was used to develop a genetic map for *C. sativa*. The newly developed GoldenGate array was hybridized with DNA from the two parental lines and 180 RI lines. Eighteen of the probes on the array gave poor signals with normalized *R* values <0.2 for each sample. Two hundred and seven probes on the array showed no polymorphism between the parental lines. The majority of these monomorphic loci (189) were designed from the 3′ cDNA data, and only 18 of these loci had been designed to specifically target SNP variation between Lindo and Licalla. The cluster distribution for the remaining probes on the array varied in pattern and ease of scoring (Fig. 1). The majority of the SNP assays showed a pattern that was distinguished by three clearly defined clusters representing the three genotypes in the mapping population (Fig. 1a). In some instances, although three clusters were observed, one allele was far less tightly clustered than its counterpart suggesting perhaps additional SNP variation in the flanking DNA could be impacting the efficacy of the hybridization (Fig. 1b). In rare cases both alleles showed loose clustering indicating poor hybridization. Such anomalies could in extreme cases suggest additional clusters; however, mapping of the loci showed normal segregation was occurring. Differences in separation of the clusters was also observed and in some cases the variance in normalized theta value between the two alleles was extremely small, requiring manual cluster calling in the GenomeStudio software (Fig. 1c). A very small subset of SNP loci (7) appeared to be dominant in nature, with only one of the alleles showing significant fluorescence levels (normalized R values). For such loci determination of heterozygous individuals was not possible (Fig. 1d).

**Fig. 2:**
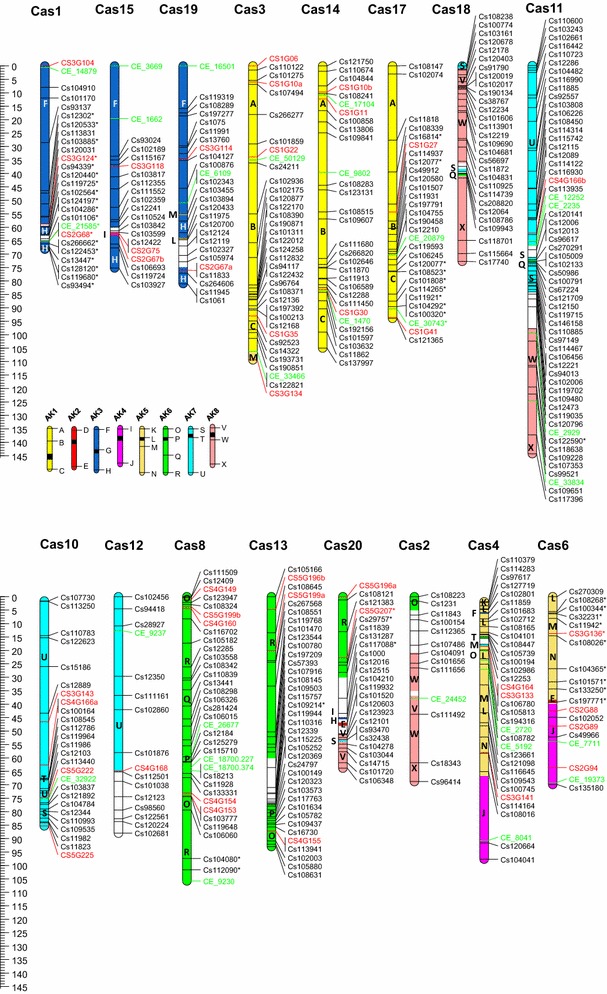

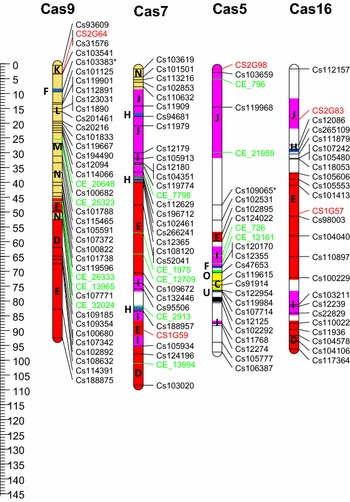
Genetic linkage map of twenty chromosomes (Cas1-20) of *C. sativa*. SNP loci (locus names have been shortened for brevity) are indicated in *black* (reduced representation of genomic DNA) and *green* (3′ cDNA); additional SSR loci are indicated in *red*. The ancestral blocks are indicated by *colour* of AK chromosome of origin and by letter (A–X). *Asterisks* to right of locus name indicate significant segregation distortion (*p* < 0.01). (Color figure online)

After manual editing of the GenomeStudio cluster file it was possible to score and map 533 SNP loci. These were arranged over twenty linkage groups, representing the haploid chromosome number of *C. sativa* (Table 1; Fig. 2). Forty-six EST-SSR loci that had previously been mapped on 90 lines of the same population were added to give a final genetic map composed of 579 loci distributed over 1,808.7 cM. There were at least 4 instances where significant (>20 cM) gaps in the linkage map (Cas 4, 15, 17 and 18) were observed. These regions were not associated with the four regions where segregation ratios for multiple linked loci were significantly (*p* < 0.01) imbalanced (Cas 1, 6, 17 and 20).

**Table 1:** Genetic linkage map of *Camelina sativa*

*C. sativa* linkage group	No. of SNP loci	No. of SSR loci	Total loci	cM	Average distance between loci (cM)	Average distance between loci (Mb)^1^
Cas1	23	3	26	68.2	2.62	0.84
Cas2	13	0	13	68.8	5.29	1.96
Cas3	31	5	36	109.4	3.04	0.70
Cas4	31	3	34	95.8	2.82	0.73
Cas5	24	1	25	95.5	3.82	1.28
Cas6	15	4	19	69.9	3.68	1.14
Cas7	32	1	33	106.7	3.23	0.96
Cas8	30	5	35	105.8	3.02	0.77
Cas9	38	1	39	92.0	2.36	0.88
Cas10	22	4	26	83.7	3.22	0.95
Cas11	57	1	58	145.8	2.51	0.80
Cas12	15	1	16	88.0	5.5	1.72
Cas13	34	3	37	93.0	2.51	0.60
Cas14	27	3	30	105.0	3.5	0.99
Cas15	17	3	20	75.2	3.76	1.33
Cas16	22	2	24	94.5	3.94	1.09
Cas17	27	2	29	93.9	3.24	1.05
Cas18	29	0	29	72.7	2.51	0.71
Cas19	24	2	26	81.0	3.11	0.95
Cas20	22	2	24	63.8	2.66	1.04
Total	533	46	579	1,808.7	3.12	0.95

^1^The physical position in the genome was defined based on BLAT alignment of the flanking sequence for each SNP or SSR marker

### Anchoring to the *Camelina sativa* genome and delineation of the Brassicaceae ancestral blocks

The 100 bp sequences flanking the SNP loci were aligned to the *C. sativa* genome sequence using BLAT (Kent 2002) with default parameters. In addition, sequences of the contigs from which each of the mapped SNP markers was derived were aligned to both the *C. sativa* and the *A. thaliana* genome using BLASTN (1E−12) (Supplementary Table 4). Similarly the EST sequences used to design the SSR primer sequences were compared to the two genomes. There was a strong correlation between the genetic and physical maps of *C. sativa* (Supplementary Figure 1); however, in regions of reduced recombination there were minor discrepancies between the marker order of the genetic map and the genome sequence. On average the markers were distributed 1 locus per 1 Mb of genome sequence, the regions with increased recombination or the larger gaps in the map corresponded to a paucity of loci selected for the particular genomic segment with physical distances ranging from 3.7 to 6.2 Mb between the loci. Some of the centromeric regions also displayed a low density of SNP loci, which was not reflected in the genetic distance (Supplementary Table 4).

Comparative alignment of 413 loci with homology either to *A. thaliana* genes or adjacent genome sequence identified the Brassicaceae ancestral blocks (A–X) defined by Schranz et al. (2006) (Supplementary Table 5; Fig. 2). These alignments were subsequently confirmed through the comprehensive analyses offered by alignment of the *C. sativa* genome sequence with the *A. thaliana* genome in Kagale et al. (2014). The SNP loci allow delineation of shared ancestry across the Brassicaceae, which assists with the identification of candidate genes underlying genomic regions of interest, in particular providing access to the extensive annotation of the *A. thaliana* genome.

### Genetic variation among *C.sativa* accessions

The newly developed *C. sativa* SNP array was used to genotype 178 *C. sativa* accessions, three lines had >20 % missing values and were excluded from further analyses. The cluster patterns observed for the SNP loci were similar to those observed for the mapping population, although further clusters were observed in some instances presumably due to the presence of additional SNP variation in the DNA flanking the SNP position found among the diversity collection. Based on automated calling 232 of the 768 SNPs were uninformative, and 11 had >20 % missing genotype values; thus 493 SNP loci were used for further analyses. Basic information including PIC value (ranging from 0.006 to 0.375), gene diversity (0.006–0.5) and major allele frequency (0.5–0.99) for each SNP locus is provided in Supplementary Table 6. The gene diversity for the entire collection was 0.26, which is lower than a similar analysis of elite maize germplasm (Van Inghelandt et al. 2010). A recent study by Delourme et al. (2013) which assessed SNP variation among germplasm of the related allotetraploid *Brassica napus* presented PIC values as a measure of gene diversity for each SNP locus. In comparing mean PIC values between the species invariably lower PIC values were seen for *C. sativa*, where values for each linkage group ranged from 0.153 to 0.286 in *C. sativa* and from 0.292 to 0.330 in *B. napus* (Supplementary Table 7). A very high inbreeding coefficient (*F*_IS_ value) of 0.96 was calculated from the *C. sativa* lines that can be explained by the inbreeding nature of the species whereas the overall fixation index (*F*_ST_ value) of 0.276, which provides a measure of population differentiation, indicates a similar level of differentiation among sub-populations as that found among winter and spring types of *B. napus* (Delourme et al. 2013).

**Fig. 3:**
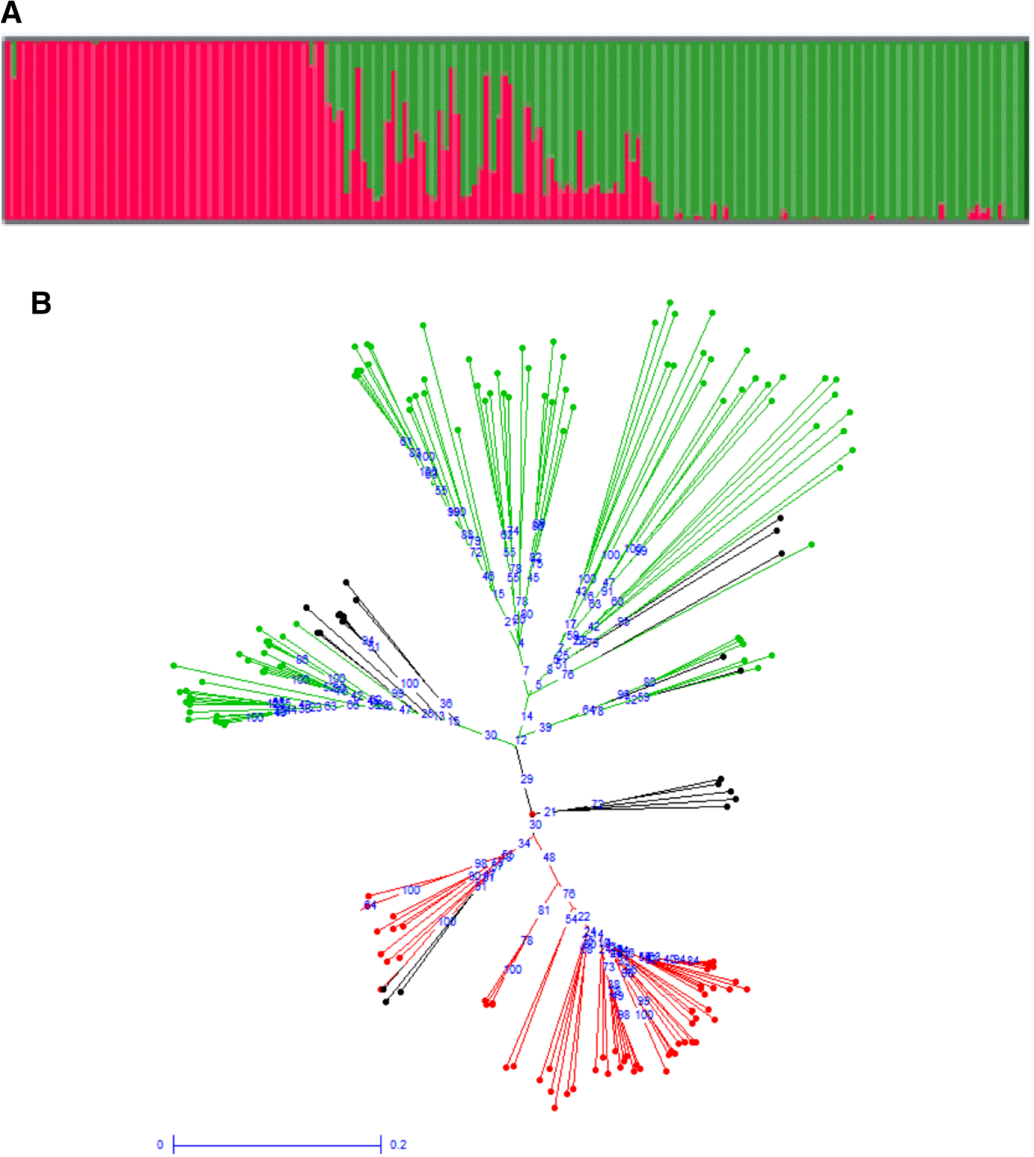
Patterns of molecular variation in 175 *C. sativa* accessions. **a** STRUCTURE analyses showing population membership of each line (*y*-axis) based on *Q* value (*x*-axis) indicated in *red* (population 1) and *green* (population 2). **b** Phylogenetic relationship among 175 *C. sativa* accessions based on the unweighted neighbour joining method. (Color figure online)

Population structure analysis was completed using STRUCTURE (Pritchard et al. 2000) for 175 accessions. Since the estimated log-likelihood values appeared to be an increasing function of *K* for all examined values of *K*, inferring the exact value of *K* was not straightforward (Supplementary Figure 2a). Using the program Structure Harvester (Evanno et al. 2005) maximal ∆*K* revealed that at a *K* value of 2 the accessions were clustered into two sub-populations (Supplementary Figure 2b). Using a minimum value of 70 % ancestry, 152 accessions were assigned to one of the two sub-populations, 61 accessions to Population I and 91 accessions to Population II (Fig. 3a). The remaining 23 accessions appeared to be admixtures or have ancestry from more than one population, with *qK* values <70 % for both populations (Supplementary Table 1). The population clusters did not group according to the available geographical information. A similar pattern was observed for the relationship as determined by the unweighted Neighbour-Joining method, which clustered accessions into two major groups. In Fig. 3b, the red and green branches on the tree represent Populations I and II, respectively as determined by STRUCTURE; all accessions defined as admixtures are shown in black. Similar to the STRUCTURE analysis, the resultant phylogenetic tree did not cluster the accessions based on geographical origin, with the lines derived from each country being evenly distributed between the populations.

## Discussion

The recent resurgence of interest in *C. sativa* as a feedstock for the bioproducts industry (Eynck and Falk 2013) has led to significant advances in the development of resources, which begin to rival those available for its *Brassica* crop relatives. The recent publication of a genome sequence for *C. sativa* provides a clear picture of the hexaploid genome structure and will be a foundational resource for genetic manipulation of the crop (Kagale et al. 2014). However, basic tools for crop improvement are still required, such as robust, high-throughput molecular markers for marker-assisted breeding. Two alternative approaches to the development of SNP markers for *C. sativa* were applied to the species prior to the availability of the genome sequence and their efficacy tested through genetic mapping and by assessing available molecular variation in a public germplasm collection.

The two approaches for SNP discovery utilized next generation sequencing technologies combined with genomic reduction methods, one targeting expressed sequences and the second genomic DNA. The first approach exploits the knowledge that sequence variation is greater in untranslated regions of transcripts, and by targeting the 3′ end of the transcript enhances the captured sequence depth, which improves the efficacy of SNP discovery (Eveland et al. 2008; Parkin et al. 2010; Koepke et al. 2012). The second approach used a simple genome reduction technique, whereby digestion with a single 6 bp recognition restriction enzyme is followed by size selection to limit genome coverage (Young et al. 2010). Both approaches proved effective in identifying SNP variants; however, the more normal distribution of read coverage from the genomic DNA and the greater depth offered by the Illumina platform led to higher numbers of SNP variants being detected with the genomic reduction method. Gehringer et al. (2006) developed the mapping population used in this study and suggested the skewed segregation pattern they observed for 21 % of AFLP loci, which were excluded from the map, and the fact that 56 % of the SSR primers amplified multiple loci, resulted from the presence of duplicated loci due to the underlying polyploidy in the *C. sativa* genome. This is a common problem faced in the design of molecular markers for polyploid species, where any amplification or hybridization based marker will invariably assay multiple orthologous or paralogous sequences (Dufresne et al. 2014). The recent publication of the *C. sativa* genome sequence (Kagale et al. 2014) demonstrated the high level of gene and genome redundancy in this species, with limited gene fractionation after the foundation of the hexaploid genome. In designing the SNP assays, the polyploid nature of the *C. sativa* genome necessitated more stringent post-discovery screening of SNP loci to reduce the likelihood of designing assays to such inter-paralogue variation. It is common to allow between 10 and 30 % variance for allele calls within SNP discovery pipelines, allowing for sequencing errors or misalignment of sequence reads; however, in the current study only SNPs called with zero variance in either the de novo assembled reference or aligned reads were selected for assay design. This necessarily limited the number of available SNP loci reducing the level of variation to 5–20 % of the total observed. However, 97 % of the SNP assays designed specifically to the parents of the recombinant inbred population were successfully mapped with limited evidence of significant segregation distortion, indicating the efficiency of the design approach for polyploid genomes.

The use of inferred collinearity with *A. thaliana* allowed the selection of SNP loci distributed relatively evenly across the *C. sativa* genome. The resultant genetic map spanned all of the expected 20 linkage groups, with a SNP locus found on average every 3.4 cM with only a small number of significant gaps (>20 cM) that equated to relatively large physical distances indicating a paucity of markers in these regions. The linkage groups ranged from 63.8 cM (Cas20) to 145.8 cM (Cas11) and together covered 1,808.7 cM. This was somewhat larger than the previously published map (1,385.6 cM) for the same mapping population (Gehringer et al. 2006), probably due to the considerably higher marker density and greater coverage of the genome. Alignment of the sequenced contigs to the *C. sativa* genome sequence (Kagale et al. 2014) anchored the developed map to the physical sequence providing a direct link to regions for targeted marker design and to the identification of candidate genes controlling traits of interest.

Only 17.5 % of the SNP loci designed from the 3′ cDNA sequences were polymorphic between the parents of the mapping population although 45.3 % were informative in assessing genetic diversity across the wider *C. sativa* germplasm collection. Although the use of transcriptome sequence for SNP discovery has the advantage of intrinsic complexity reduction, such data can be complex to mine for variation due to biased representation resulting from the nuances of gene expression and the inherent redundancy arising from gene duplication (Ganal et al. 2009). Therefore it was perhaps predictable that in comparison to the 3′ cDNA SNP loci, almost double the number of SNPs (79.1 %) designed from the genomic DNA were informative across the *C. sativa* accessions. Similar to previous genetic diversity analyses carried out on smaller collections of *C. sativa* (Manca et al. 2013; Vollmann et al. 2005), two well-differentiated populations could be identified among the germplasm investigated in the present study. Population stratification revealed by molecular diversity studies in plants can be a consequence of a number of factors, including mating habit, geographic origin, environmental selection pressure, migration and in the case of crop plants—human selection or domestication (Dufresne et al. 2014). Currently we have limited knowledge of the history or origin of *C. sativa* and as with the previous studies neither the phylogenetic tree nor the STRUCTURE results clustered the accessions based on the expected geographical distribution. This could be due to unresolved conflicts between the actual origin of an accession and the country that donated the accession to the genebank. There is no suggestion of differential mating habits among the *C. sativa* accessions studied. Furthermore, the strong inbreeding nature of *C. sativa*, which reduces the effective population size, could lead to rapid isolation of a sub-population that has a selective advantage. It is interesting to speculate that the spread of *C. sativa* from Europe to North America, possibly as a contaminant of flax seed, may have contributed to the current population structure; however, further work will be needed to characterise the observed differentiation (Francis and Warwick 2009). The estimate of genetic variability provided by PIC value as a measure of gene diversity is low (0.224 for mapped loci) when compared to an analyses of the related crop species *B. napus* (0.310) described by Delourme et al. (2013). Although there was variation for PIC value both among *C. sativa* linkage groups and along their lengths (Supplementary Table 7, Supplementary Figure 3) as observed for *B. napus*, there were no significant differences found in mean PIC value either between the triplicated sub-genomes or when independently analysing the sub-populations, whereas differences were observed both between sub-genomes and across morphotypes in *B. napus* (Delourme et al. 2013). Again this probably reflects the limited breeding pressure to which *C. sativa* has been exposed. Although variation has been identified for a number of phenotypic traits of value, including oil profiles and downy mildew resistance (Vollmann et al. 2001, 2007), the relatively low gene diversity of *C. sativa* combined with the complexities of working with a hexaploid could prove frustrating for breeding programs targeting this novel oilseed. It maybe that alternative approaches to manipulating the genome, such as simultaneously manipulating entire gene families would be more promising (Nguyen et al. 2013).

The current study exploited reduction representation and NGS to carry out SNP discovery for the hexaploid genome of *C. sativa*. A comparison of transcriptome and genomic targets suggested the latter were more efficient substrates for developing robust markers, in particular for a polyploid genome that requires additional filtering of potential SNP variants. Although designed from only four genotypes, the developed Illumina GoldenGate SNP assays showed sufficient polymorphism for molecular characterization and genetic diversity analyses in a large collection of *C. sativa* accessions. This SNP genetic map of *C. sativa* provides an important tool for navigation from trait loci to the recently published genome sequence. Furthermore, the current assays can be readily converted for use on other platforms. Hence they represent an important resource for genetic characterization of additional mapping populations and can be readily applied in current breeding programs.

## Electronic supplementary material

Below is the link to the electronic supplementary material.

Supplementary material 1 (XLSX 176 kb)

Supplementary material 2 (PDF 491 kb)
