# Supplementary material for: Single-nucleotide polymorphism identification and genotyping in Camelina sativa
Source: Mol Breed. 2015 Jan 21;35(1):35. doi: 10.1007/s11032-015-0224-6 (PMC4300397; doi:10.1007/s11032-015-0224-6)
Supplement: Supplementary file 2 — Supplementary material 2 (PDF 491 kb) [file 11032_2015_224_MOESM2_ESM.pdf]

Supplementary Figure S1: Plot of genetic map position (cM) on x-axis against physical map position (Mb) on the y-axis for each SNP marker on the *C. sativa* genetic map.

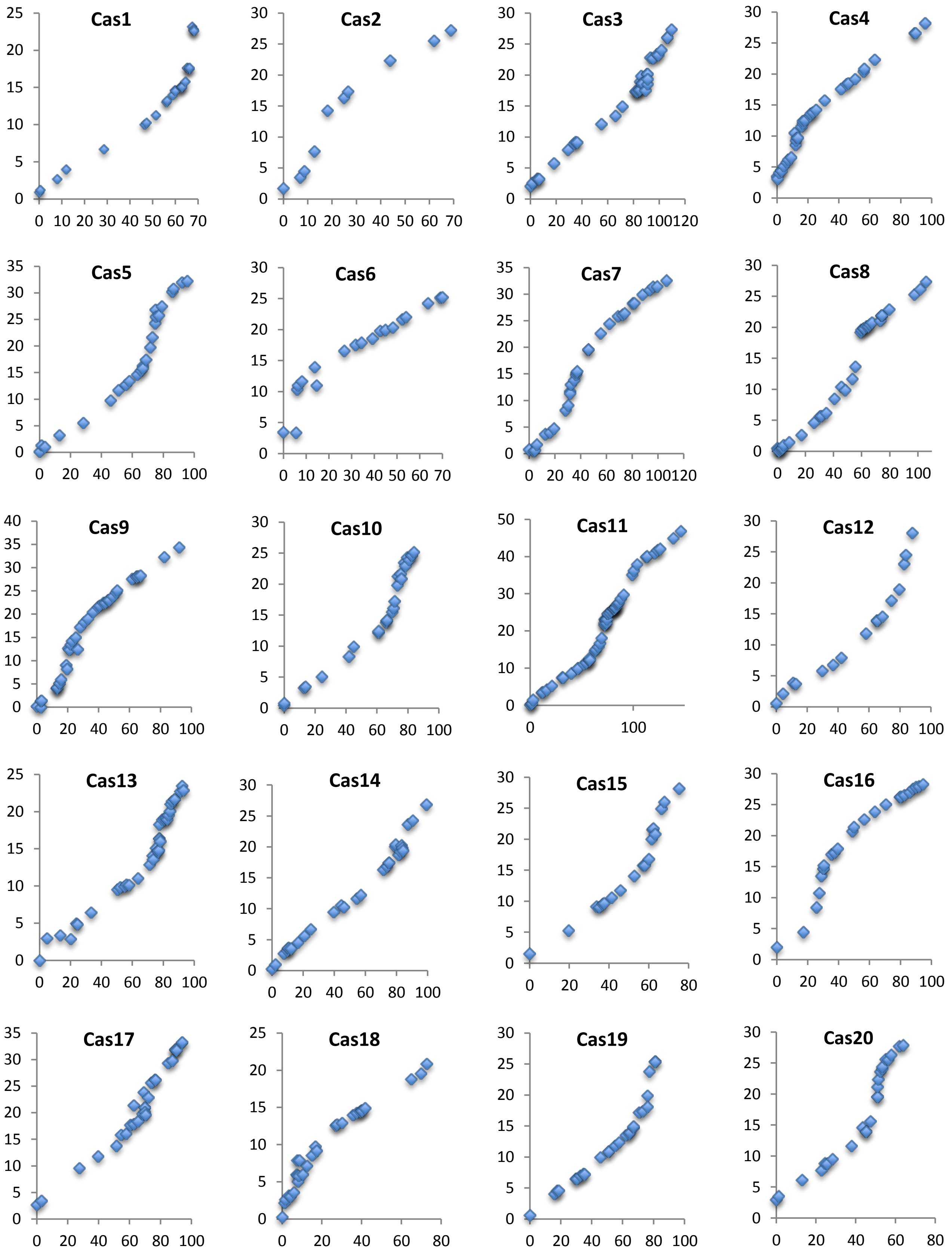

Supplementary Figure S2: Population structure analysis of 175 *C. sativa* accessions by STRUCTURE. (a) Estimated LnP(D) of possible clusters (k) from1to 10; (b) Evanno's Δk based on the rate of change of LnP(D) between successive k.

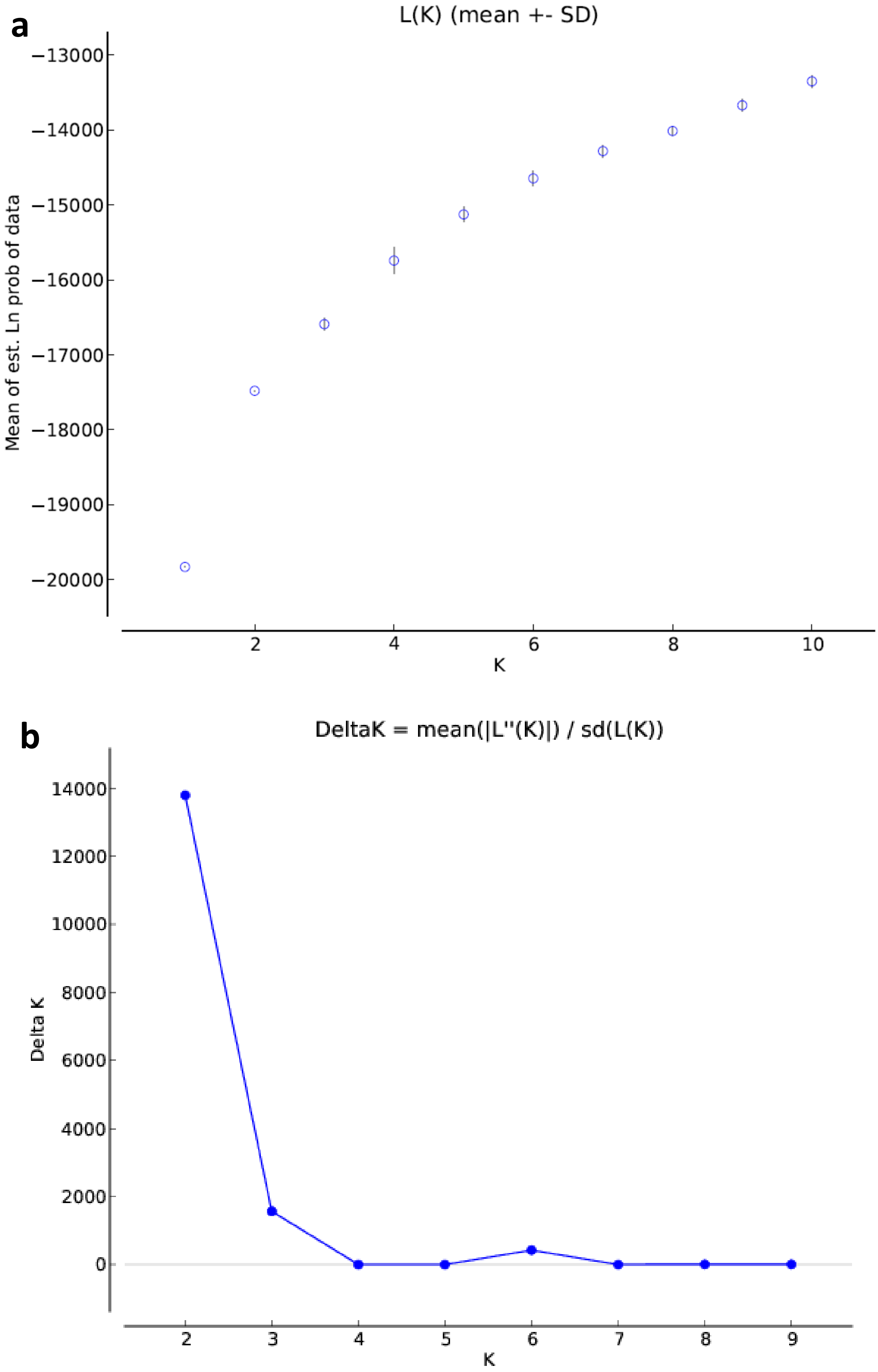

Supplementary Figure S3: Distribution of PIC values along the *C. sativa* linkage groups in the sub-populations K1, K2 and admixture groups along with whole collection of *C. sativa* accessions.

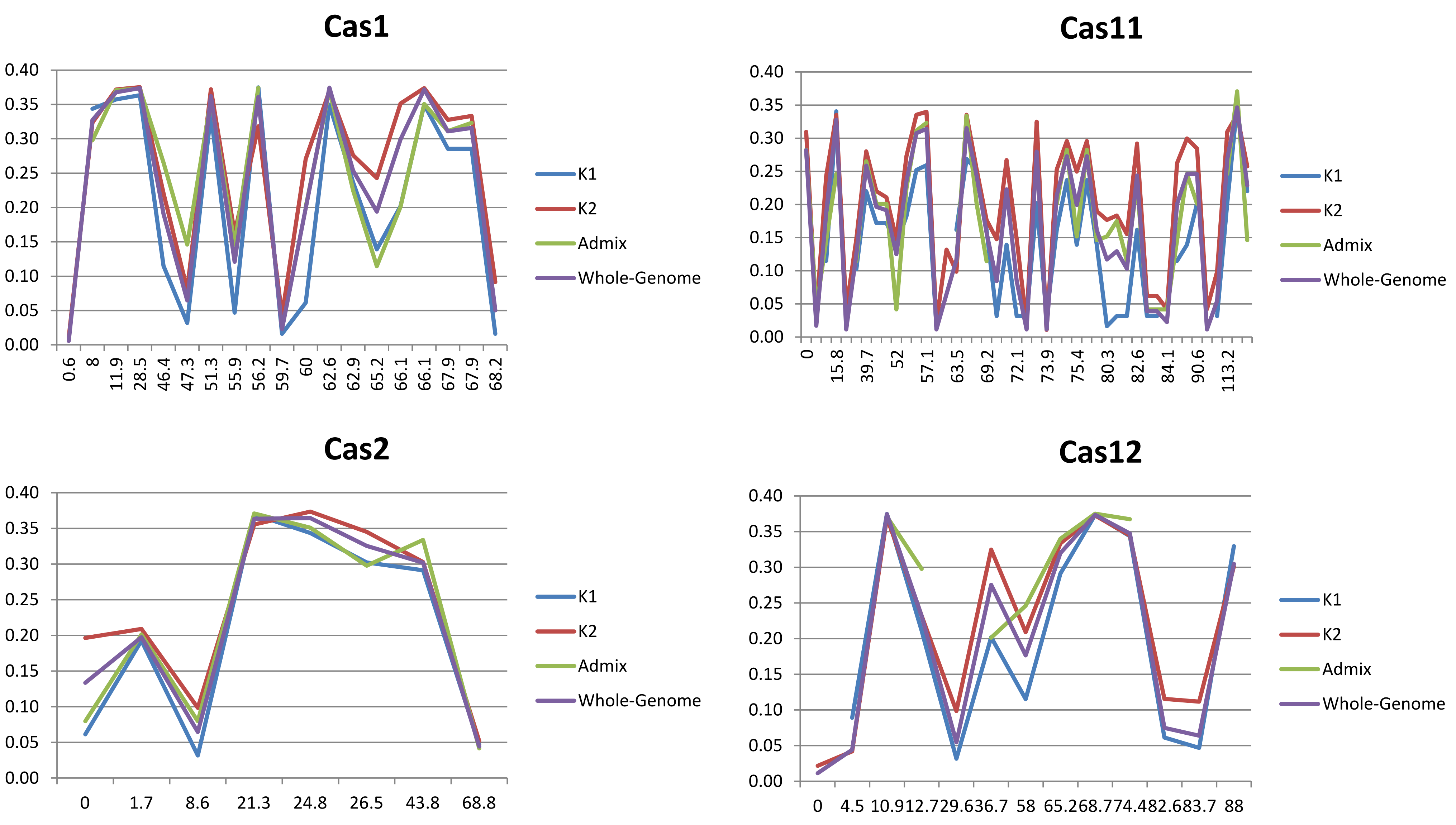

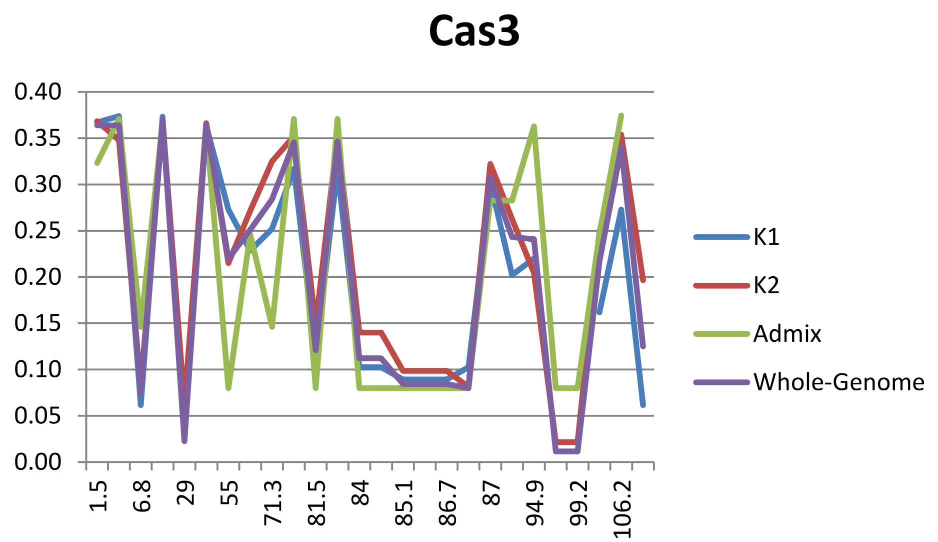
Cas3
0.40
0.35
0.30
0.25
0.20
0.15
0.10
0.05
0.00
1.5
6.8
29
55
71.3
81.5
84
85.1
86.7
87
94.9
99.2
106.2
K1
K2
Admix
Whole-Genome

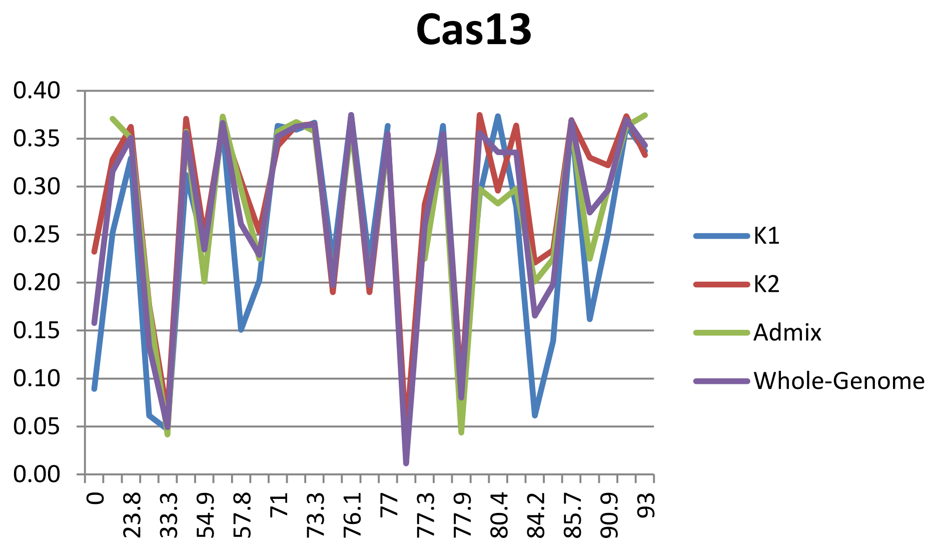
Cas13
0.40
0.35
0.30
0.25
0.20
0.15
0.10
0.05
0.00
0
23.8
33.3
54.9
57.8
71
73.3
76.1
77
77.3
77.9
80.4
84.2
85.7
90.9
93
K1
K2
Admix
Whole-Genome

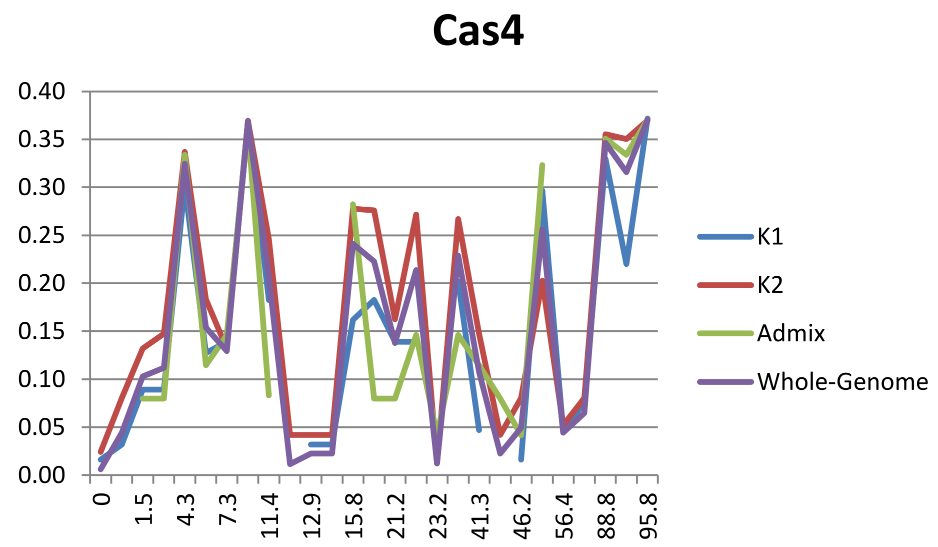
Cas4
0.40
0.35
0.30
0.25
0.20
0.15
0.10
0.05
0.00
0
1.5
4.3
7.3
11.4
12.9
15.8
21.2
23.2
41.3
46.2
56.4
88.8
95.8
K1
K2
Admix
Whole-Genome

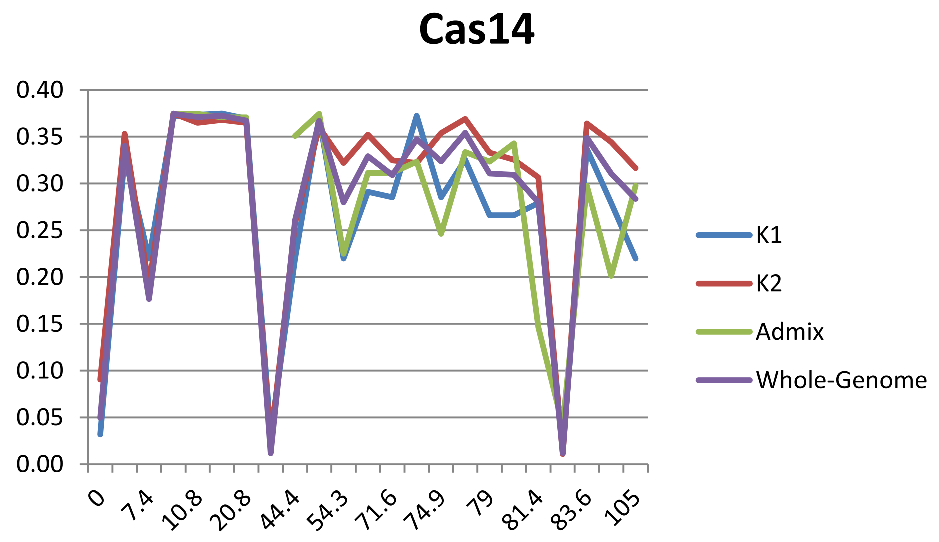
Cas14
0.40
0.35
0.30
0.25
0.20
0.15
0.10
0.05
0.00
0
7.4
10.8
20.8
44.4
54.3
71.6
74.9
79
81.4
83.6
105
K1
K2
Admix
Whole-Genome

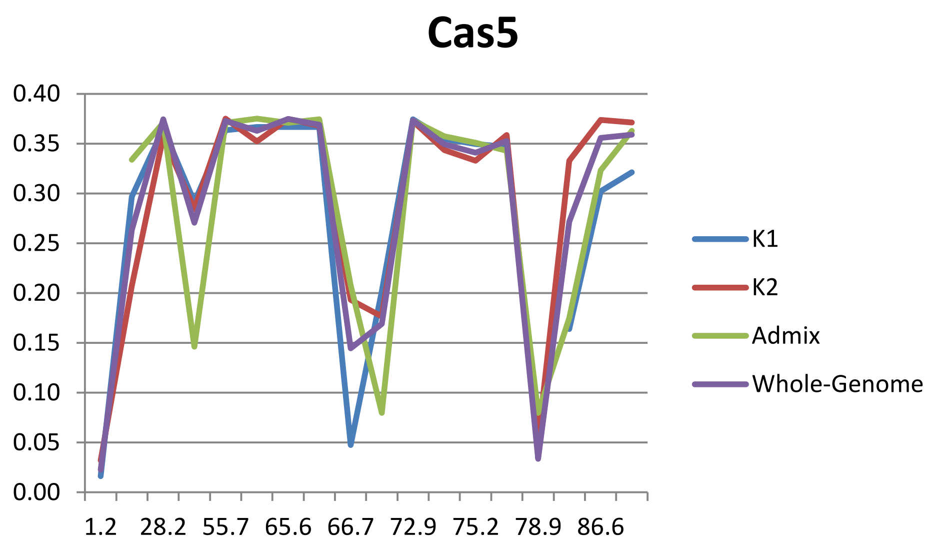
Cas5
0.40
0.35
0.30
0.25
0.20
0.15
0.10
0.05
0.00
1.2
28.2
55.7
65.6
66.7
72.9
75.2
78.9
86.6
K1
K2
Admix
Whole-Genome

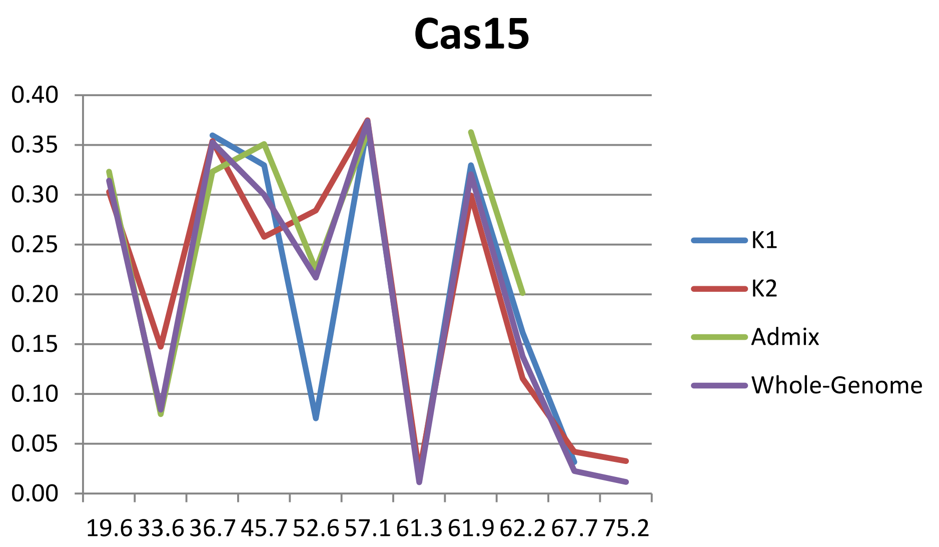
Cas15
0.40
0.35
0.30
0.25
0.20
0.15
0.10
0.05
0.00
19.6
33.6
36.7
45.7
52.6
57.1
61.3
61.9
62.2
67.7
75.2
K1
K2
Admix
Whole-Genome

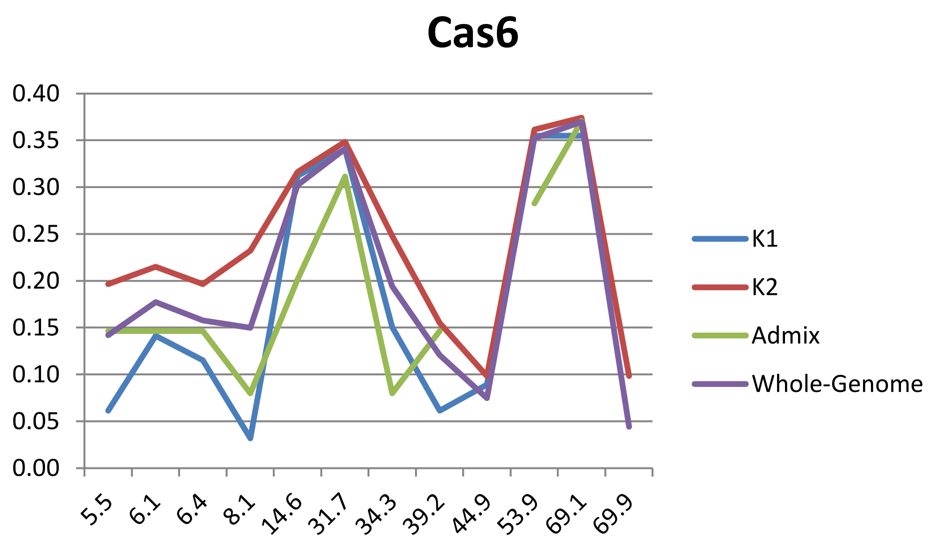
Cas6
0.40
0.35
0.30
0.25
0.20
0.15
0.10
0.05
0.00
5.5
6.1
6.4
8.1
14.6
31.7
34.3
39.2
44.9
53.9
69.1
69.9
K1
K2
Admix
Whole-Genome

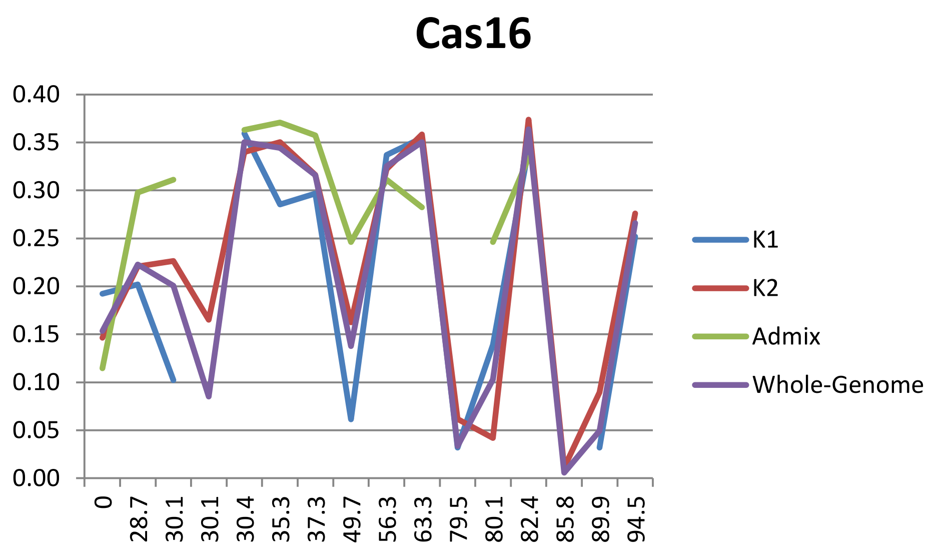
Cas16
0.40
0.35
0.30
0.25
0.20
0.15
0.10
0.05
0.00
0
28.7
30.1
30.1
30.4
35.3
37.3
49.7
56.3
63.3
79.5
80.1
82.4
85.8
89.9
94.5
K1
K2
Admix
Whole-Genome

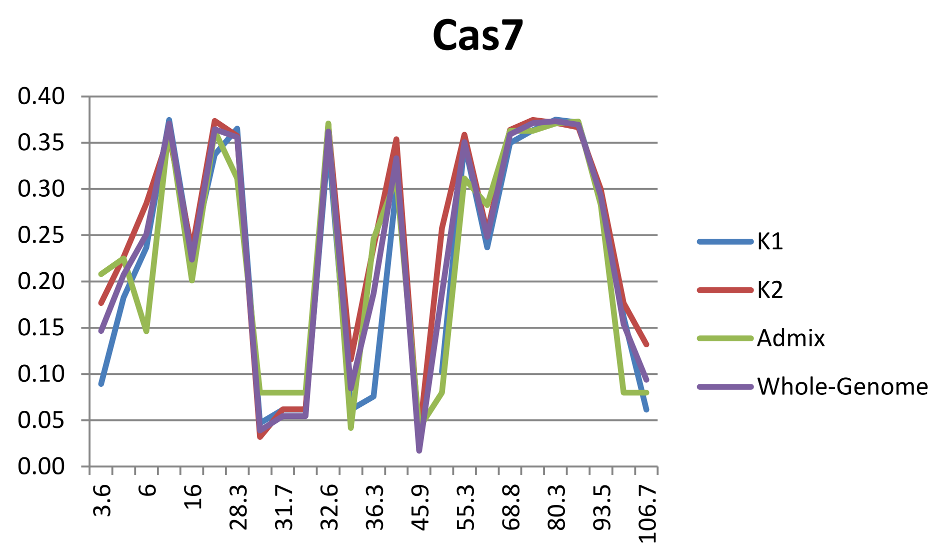
Cas7
0.40
0.35
0.30
0.25
0.20
0.15
0.10
0.05
0.00
3.6
6
16
28.3
31.7
32.6
36.3
45.9
55.3
68.8
80.3
93.5
106.7
K1
K2
Admix
Whole-Genome

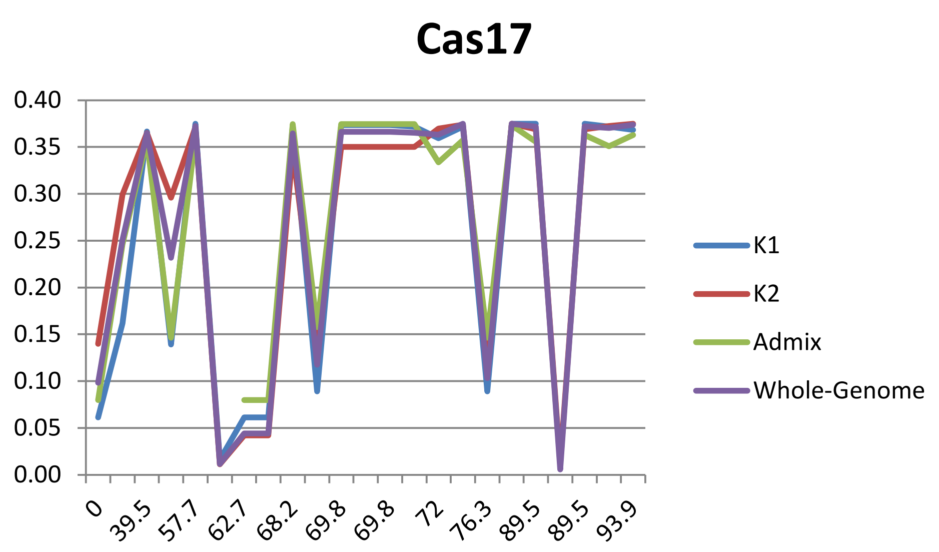
Cas17
0.40
0.35
0.30
0.25
0.20
0.15
0.10
0.05
0.00
0
39.5
57.7
62.7
68.2
69.8
69.8
72
76.3
89.5
89.5
93.9
K1
K2
Admix
Whole-Genome

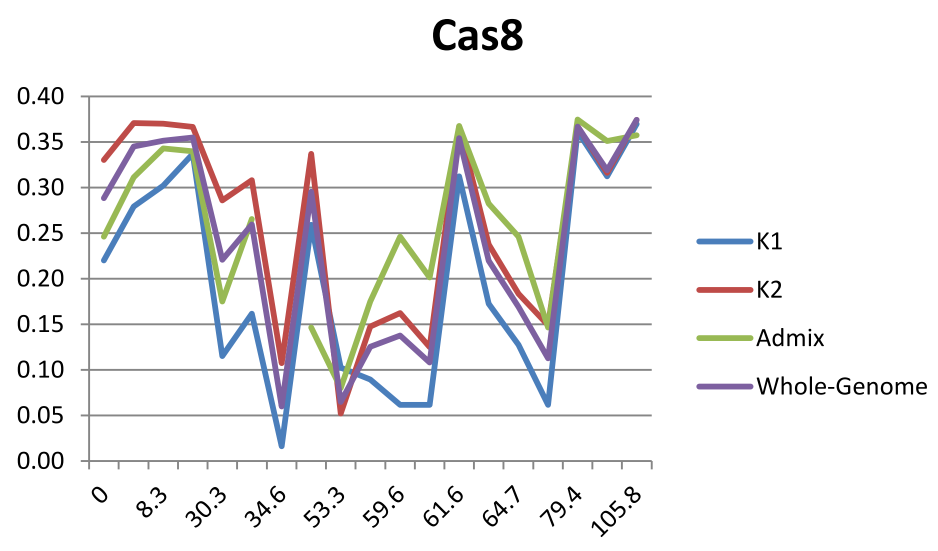
Cas8
0.40
0.35
0.30
0.25
0.20
0.15
0.10
0.05
0.00
0
8.3
30.3
34.6
53.3
59.6
61.6
64.7
79.4
105.8
K1
K2
Admix
Whole-Genome

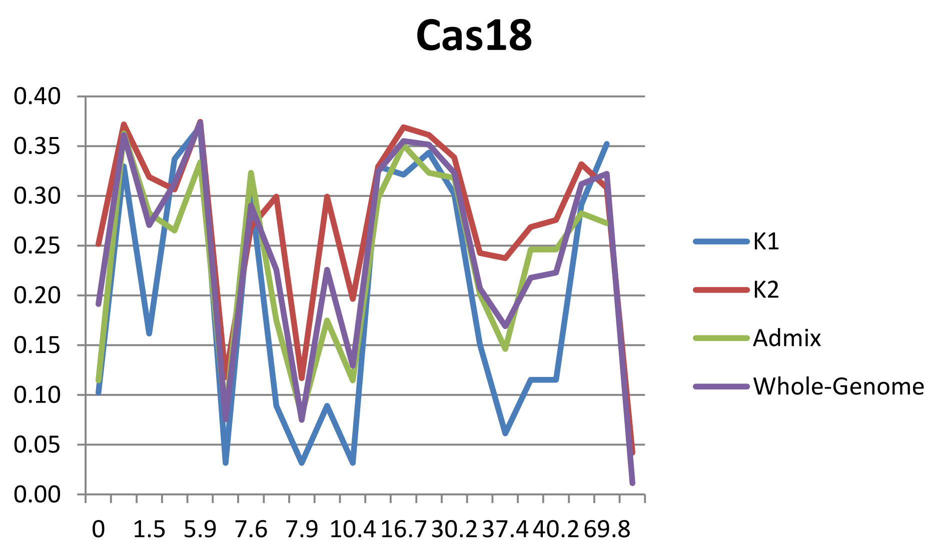
Cas18
0.40
0.35
0.30
0.25
0.20
0.15
0.10
0.05
0.00
0
1.5
5.9
7.6
7.9
10.4
16.7
30.2
37.4
40.2
69.8
K1
K2
Admix
Whole-Genome

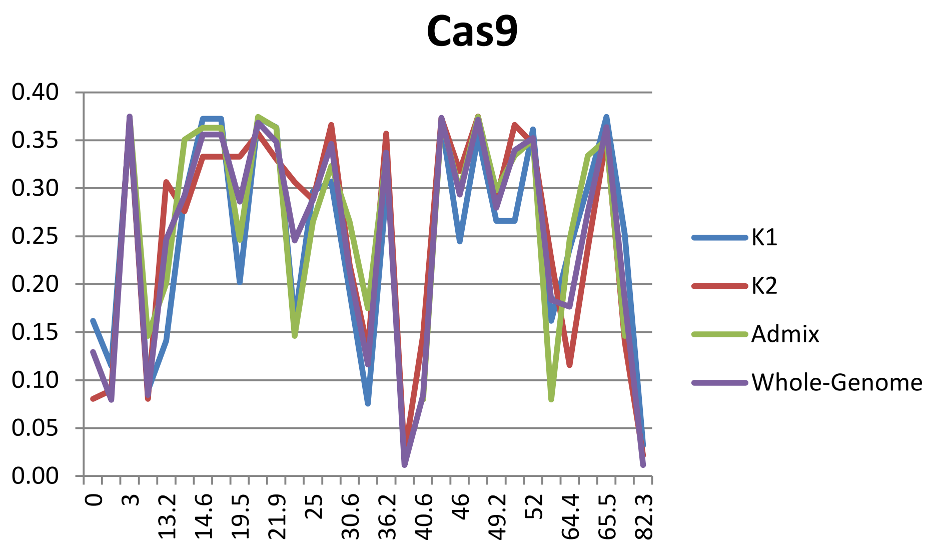
Cas9
0.40
0.35
0.30
0.25
0.20
0.15
0.10
0.05
0.00
0
3
13.2
14.6
19.5
21.9
25
30.6
36.2
40.6
46
49.2
52
64.4
65.5
82.3
K1
K2
Admix
Whole-Genome

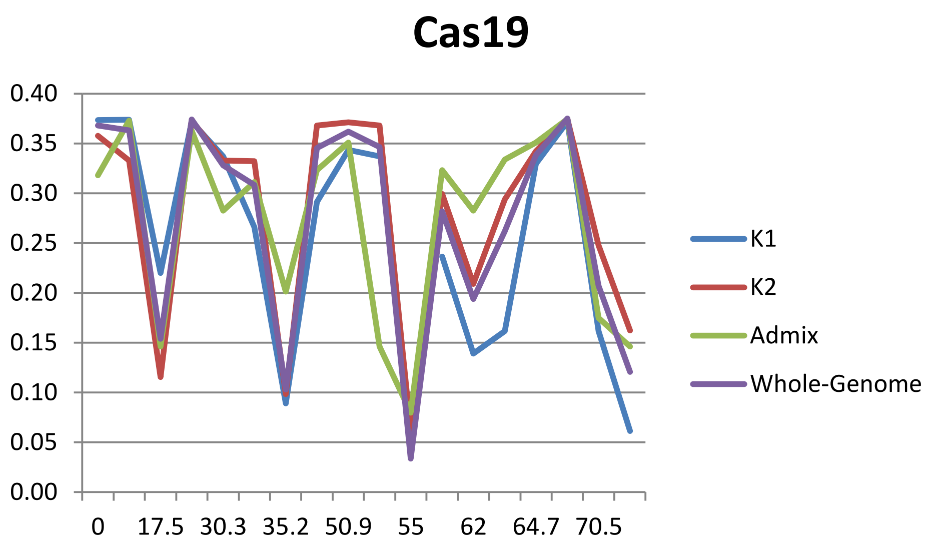
Cas19
0.40
0.35
0.30
0.25
0.20
0.15
0.10
0.05
0.00
0
17.5
30.3
35.2
50.9
55
62
64.7
70.5
K1
K2
Admix
Whole-Genome

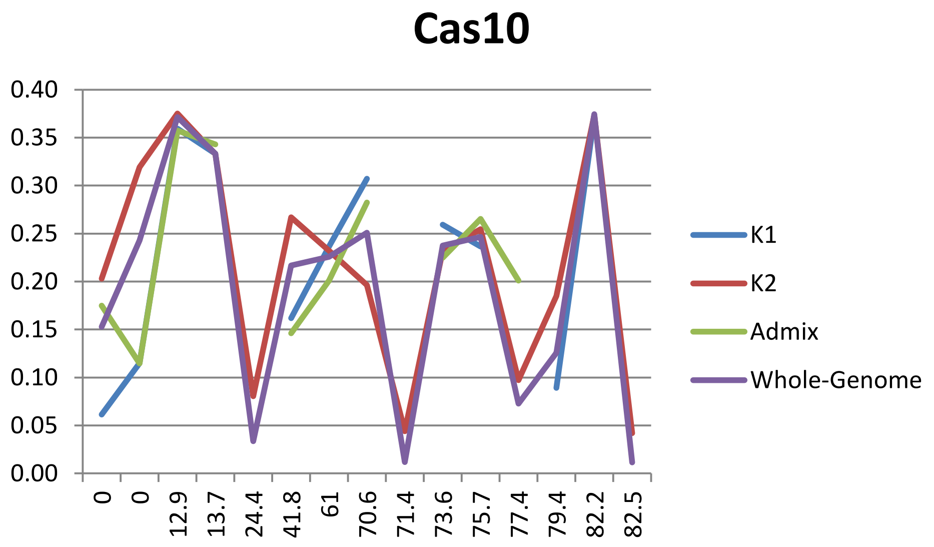
Cas10
0.40
0.35
0.30
0.25
0.20
0.15
0.10
0.05
0.00
0
0
12.9
13.7
24.4
41.8
61
70.6
71.4
73.6
75.7
77.4
79.4
82.2
82.5
K1
K2
Admix
Whole-Genome

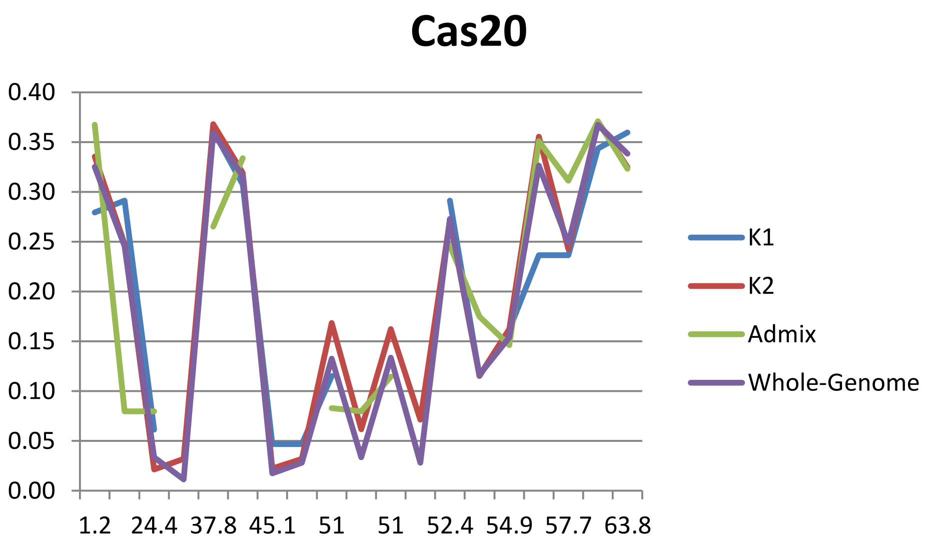
Cas20
0.40
0.35
0.30
0.25
0.20
0.15
0.10
0.05
0.00
1.2
24.4
37.8
45.1
51
51
52.4
54.9
57.7
63.8
K1
K2
Admix
Whole-Genome
